# Generalization of Dexterous Manipulation Is Sensitive to the Frame of Reference in Which It Is Learned

**DOI:** 10.1371/journal.pone.0138258

**Published:** 2015-09-16

**Authors:** Michelle Marneweck, Elisabeth Knelange, Trevor Lee-Miller, Marco Santello, Andrew M. Gordon

**Affiliations:** 1 Department of Biobehavioral Sciences, Teachers College, Columbia University, New York, New York, United States of America; 2 MOVE Research Institute, Faculty of Human Movement Sciences, Vrije Universiteit Amsterdam, Amsterdam, North Holland, The Netherlands; 3 School of Biological and Health Systems Engineering, Arizona State University, Tempe, Arizona, United States of America; The University of Western Ontario, CANADA

## Abstract

Studies have shown that internal representations of manipulations of objects with asymmetric mass distributions that are generated within a specific orientation are not generalizable to novel orientations, i.e., subjects fail to prevent object roll on their first grasp-lift attempt of the object following 180° object rotation. This suggests that representations of these manipulations are specific to the reference frame in which they are formed. However, it is unknown whether that reference frame is specific to the hand, the body, or both, because rotating the object 180° modifies the relation between object and body as well as object and hand. An alternative, untested explanation for the above failure to generalize learned manipulations is that any rotation will disrupt grasp performance, regardless if the reference frame in which the manipulation was learned is maintained or modified. We examined the effect of rotations that (1) maintain and (2) modify relations between object and body, and object and hand, on the generalizability of learned two-digit manipulation of an object with an asymmetric mass distribution. Following rotations that maintained the relation between object and body and object and hand (e.g., rotating the object and subject 180°), subjects continued to use appropriate digit placement and load force distributions, thus generating sufficient compensatory moments to minimize object roll. In contrast, following rotations that modified the relation between (1) object and hand (e.g. rotating the hand around to the opposite object side), (2) object and body (e.g. rotating subject and hand 180°), or (3) both (e.g. rotating the subject 180°), subjects used the same, yet inappropriate digit placement and load force distribution, as those used prior to the rotation. Consequently, the compensatory moments were insufficient to prevent large object rolls. These findings suggest that representations of learned manipulation of objects with asymmetric mass distributions are specific to the body- and hand-reference frames in which they were learned.

## Introduction

To skillfully manipulate objects with the fingertips requires the ability to adapt the fingertip forces to the constraints imposed by an object’s physical properties. This requires both the use of tactile feedback and feedforward (anticipatory) control, the latter of which takes advantage of the stable and predictable physical properties of objects. These feedforward control predictions are based on visual cues of the object’s properties and internal representations (sensorimotor memories) associated with the object [1,2,3]. Visual cues can provide direct information about object size [4,5,6], shape [7,8,9,10], and identity [1], but might also provide indirect information about weight and mass distribution, which can sometimes be imprecise. Since weight and mass distribution information is only available after object lift-off, internal representations formed during earlier experiences with the object are used to scale forces to the weight and mass distribution of the object [1,2,3].

To understand the nature of these internal representations, studies have used a paradigm in which the visual cues of the object are not salient [11,12,13,14,15,16,17,18]. This paradigm required subjects to grasp and lift with the index finger and the thumb a symmetrically shaped object, an inverted T-shaped object, with an asymmetric mass distribution (i.e. one side of the base of the inverted T-shaped object is heavier than the other side). The goal of the task was to minimize object roll. On their first object lift, subjects exerted symmetrical grip forces and symmetrical load forces by the index finger and the thumb, and thereby generated little or no compensatory moment countering the external torque caused by the asymmetric mass distribution, which resulted in a large object roll. However, subjects learned within just a few lifts to minimize object roll by applying more load force in the digit on the heavier side of the inverted T-shaped object. Subsequent studies removed digit placement constraints, whereby subjects could grasp and lift the inverted T-shaped object anywhere along two grasp surfaces [14,16,17,18,19,20]. Results of these studies showed that load forces were modulated in parallel with digit placement, i.e., higher digit placement on the heavier side of the object, with both digit positions and forces contributing to generating a compensatory moment to prevent roll.

Some studies have questioned the extent to which these internal representations generated within a specific object orientation are generalizable to novel orientations [11,12,13,15,18,20,21,22,23]. These studies typically required subjects to grasp and lift an object with an asymmetric mass distribution in one orientation, learn the manipulation, and then lift and grasp it in a novel orientation, e.g., following 180° rotation of the object. This rotation modifies both body and hand frames of reference from that to trials preceding the rotation. In other words, if the center of mass (CoM) is on the object’s left side before rotation, it would be toward the left side of the body and oppose the thumb during lifts with the right hand. Conversely, after rotating the object 180°, the CoM would oppose the index finger and be toward the right side of the body. After such object rotations, subjects failed to prevent object roll on their first attempt to grasp and lift the object due to inappropriate load force scaling [11,12,13,15,21] as well as inappropriate digit placement [18,20]. These findings suggest that internal representations of learned manipulations of objects with asymmetric mass distributions are specific to the frame of reference in which they were formed. However, it is unknown whether that frame of reference is specific to the hand, the body, or both, because rotating the object 180° modifies *both* the relation between the object and body *and* between the object and hand as described above.

An alternative, yet untested, explanation for the failure in transferring learned manipulation and therefore preventing roll might be that *any* modification in object orientation interferes with generalization of learned manipulation, regardless of whether the frame of reference is maintained or modified. In a pioneering study pointing to the complexity of mental rotation Shepard and Metzler [24] found an increase in time in identifying two shapes as similar with an increase in the angular difference between the two shapes. Given the complexities involved in identifying object geometry after mental rotation of an object, any kind of rotation, even that which maintains the body and hand frames (*e*.*g*. 360° rotation of object, or subject, or both), might disrupt transfer of learned manipulation of the same object in a different orientation.

We addressed two overarching aims in a set of 8 experiments. First, we examined the effect of rotations that maintain the relation between the both the object and body and object and hand. Second, we examined the effect of rotations that modify both and either of these relations on the ability to transfer learned manipulation. Based on previous findings, we hypothesized that rotations that modify *both* the relation between the object and body, *and* between object and hand, will impair performance. Results from rotations that modify either the relation between the object and body, or between object and hand, will elucidate whether the reference frame used to learn object manipulation is specific to the body, the hand, or both. For example, impaired performance after a rotation that modifies only the object to hand relation and correct performance following a rotation that modifies the object to body relation would suggest that internal representations of objects with asymmetric mass distributions are specific only to the hand reference frame in which it was learned (and not the body reference frame). Alternatively, impaired performance induced by both of these rotation types would suggest that internal representations of objects with asymmetric mass distributions are specific to both body and hand reference frames in which they were learned. Finally, should performance be disrupted also in conditions that do not modify reference frames, this would suggest that the act of rotation is what disrupts performance, and not the change in reference frame.

## Methods

### Participants

Eighty-seven right-handed healthy adults (59 females; *Median* age: 27; *Range*: 20–34) with normal or corrected-to-normal vision participated across 8 experimental conditions. We included in the main analyses 10 subjects for each experimental condition, excluding 7 subjects (see below). All subjects gave written informed consent and the Teachers College, Columbia University Institutional Review Board approved the study procedures.

### Materials and Procedures

**Fig 1:**
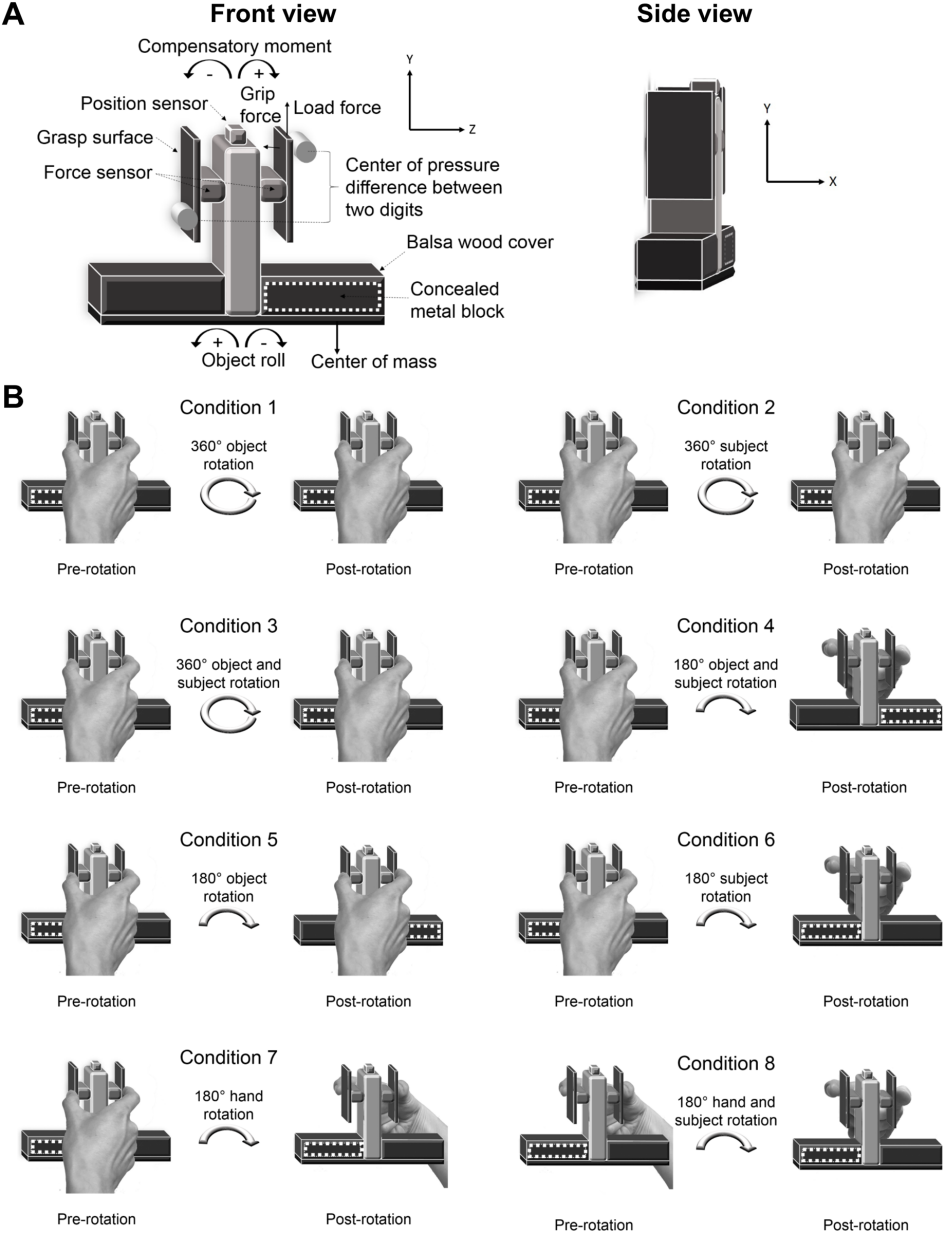
A depiction of the visually symmetrical object with a visually concealed asymmetric mass distribution, and the experimental procedure for the 8 conditions. (A) Custom built inverted T-shaped object. A solid brass metal block was placed on either the left or right side on the base of the object. The solid brass metal block was visually concealed with two balsa wood covers that were placed on the left and right side on the base of the object. Thus, the object was symmetrical in appearance but not in mass distribution. An electromagnetic position sensor was placed at the top of the vertical column to measure object roll. The grasp surfaces were attached to the force sensors that measured forces and centers of pressure of the thumb and index finger. Left and right panels show front and side views of the object; (B) Experimental procedures for each of the 8 conditions during pre-rotation trials (with the center of mass on the left; see white dotted outline) and following a rotation, the post-rotation trials. The rotation either maintained hand and body reference frames (Condition 1–4) or modified hand and/or body reference frames (Conditions 5–8). Conditions that maintained hand and body reference frames involved a 360° object rotation (Condition 1), 360° subject rotation (Condition 2), 360° object and subject rotation (Condition 3), and a 180° object and subject rotation (Condition 4). Conditions that modified hand and/or body reference frames involved a 180° object rotation (Condition 5), 180° subject rotation (Condition 6), 180° hand rotation (Condition 7), and a 180° hand and subject rotation (Condition 8). A full circle arrow indicates a 360° rotation (by object and/or subject, dependent on condition), and a half circle arrow indicates a 180° rotation (by object/and or subject and/or hand, dependent on condition).

Subjects were asked to grasp and lift an inverted T-shaped object (Fig 1A) with an asymmetric CoM using the tips of their right index finger and thumb, with the aim of preventing object roll [11,12,18,20,25].

Forces and moments exerted by the thumb and index finger were measured by two force/torque transducers (Nano 17, ATI Industrial Automation, NC). The transducers measured grip force, load force, and moment with resolutions of 0.05 N, 0.025 N, and 0.125 Nmm, respectively. The force transducers were attached parallel to each other on the vertical column of the inverted T-shaped object (made from Plexiglass). The force transducers were covered by two parallel Plexiglass grip surfaces (height: 7.0 cm; width: 1.9 cm; depth: 0.3 cm; distance between grip surfaces = 8.0 cm). Each of the Plexiglass grip surfaces was covered with a thin sheet of balsa wood to increase the friction between the digits and the object’s contact surfaces. At the base of the object, two black balsa wood surface covers visually concealed the asymmetrically distributed mass (a solid brass block; height: 2.5 cm; width: 7.0 cm; depth: 2.5 cm; mass: 405 g). Thus, the object was symmetrical in appearance (inverted “T”), but not in mass distribution. An electromagnetic position-angle sensor (Polhemus Fastrack; angular resolution: 0.025°; displacement resolution: 0.0005 cm) was attached at the top of the object to measure object roll. The total mass of the object, including the force sensors, position-angle sensor, brass, and the balsa wood covers was 585 g. Fingertip force data were sampled at 500 Hz and position data were sampled at 120 Hz using SC/Zoom (Umeå University, Sweden).

Subjects sat comfortably in front of a height-adjustable table facing the object with their right elbow flexed approximately 90° in the parasagittal plane, with their right shoulder aligned with the midpoint of the object. The right hand was placed, palm facing down, at a marked start location, 24 cm from the midpoint of the object. Following an auditory cue, subjects were instructed to reach from the marked start location, grasp the grip surfaces with the tip of the thumb and the index finger, and lift the object at a natural speed to a height of an adjacent marker (10 cm). Following a second auditory cue (occurring 5 s after first auditory cue), subjects were instructed to replace both object and hand back to their respective start locations. Subjects were asked to minimize as best possible object roll. No instruction was given regarding the location of fingertip placement on the object.

There were two blocks of 16 trials, with a 20-s inter-stimulus interval between trials. Each block contained 8 pre-rotation trials, followed by a rotation of the object, subject, subject’s hand, both object and subject, or both subject and subject’s hand (see below), and 8 post-rotation trials thereafter. Rotations were performed around the vertical axis of the object (Fig 1B). The CoM of the object was on a given side during the pre-rotation trials of the first block, and then on the other side during the pre-rotation trials of the second block. CoM location (left or right) in the first block was counterbalanced across subjects in each condition. Subjects were asked to indicate the heavier side of the object, left or right, prior to and following exposure to each rotation. Subjects (*n* = 7) that gave an incorrect response were excluded from the main analyses, but we compared their performance qualitatively to those that gave a correct response. Subjects experienced only one type of rotation, which prevented proactive interference or learning across conditions. Subjects experienced 1 of 8 experimental manipulations (*n* = 10 in each group; see Table 1 and Fig 1B) following pre-rotation trials:

**Table 1:** Description of each of the 8 experimental conditions.

Condition	Description
**Conditions that maintain both object-hand and object-body relations**	
1	360° rotation of the object
2	360° rotation of the subject
3	360° rotation of the object and subject
4	180° rotation of the object and subject
**Conditions that modify both object-hand and object-body relations**	
5	180° rotation of the object
6	180° rotation of the subject
**Condition that modifies the object-hand relation**	
7	180° rotation of the hand around the object
**Condition that modifies the object-body relation**	
8	180° rotation of the hand and subject

#### Conditions that maintain the relation between object and hand and between object and body

In Condition 1 (360° rotation of the object), subjects were asked to observe the object being rotated by the experimenter 360°. The relation between the object and body, and between the object and hand, was unchanged following this rotation, with the CoM on the same side of the body and on the same digit side during pre- and post-rotation trials.

In Condition 2 (360° rotation of the subject), subjects were instructed to stand up and walk 360° around the table and return to their pre-rotation seated position. As in Condition 1, following this rotation, the body-to-object and hand-to-object relations were the same during pre- and post-rotation trials.

In Condition 3 (360° rotation of the object and subject), subjects were asked to observe the object being rotated 360° by the experimenter and to subsequently stand up and walk in the same direction around the table and return to their pre-rotation seated position. As with Conditions 1 and 2, the relations between object and body, and between object and hand, were the same during pre- and post-rotation trials.

In Condition 4 (180° rotation of the object and subject), after observing a 180° rotation of the object by the experimenter, subjects were asked to stand up and walk 180° in the same direction around to the other side of the table, and be seated. As in Conditions 1 to 3, the object-to-body and object-to-hand relations during pre-rotation trials remained unchanged following rotation of both object and subject.

#### Conditions that modify the relation either between object and hand, between object and body, or both

In Condition 5 (180° rotation of the object), subjects were asked to observe the object being rotated 180° by the experimenter. This rotation modified the relation between the object and the subject’s body, and the relation between the object and the subject’s hand from that during pre-rotation trials. For example, for an object with a left CoM, the heavier side of the object is on the left side of the body and on the thumb side prior to object rotation. Following a 180° rotation of the object, the heavier side of the object is on the right side of the body *and* on the index finger side.

In Condition 6 (180° rotation of the subject), subjects were asked to stand up and walk 180° around to the other side of the table, and be seated facing the object from the other side of the table. Similar to the manipulation in which the object is rotated 180° (Condition 5), a 180° rotation of the subject modified the object-to-body *and* the object-to-hand relation from that during pre-rotation trials.

In Condition 7 (180° rotation of the hand around the object), subjects were asked to orient their hand 180° around the object and lift the object in this new hand configuration with the fingertips facing toward the body. This rotation modified the relation between the object and the hand, but maintained the relation between the object and the body, from that during pre-rotation trials. For example, for an object with a right CoM, the heavier side of the object is on the right side of the body before and after hand rotation, but on the index finger side before rotation and on the thumb side after rotation.

In Condition 8 (180° rotation of the hand and subject), unlike the pre-rotation trials in all other conditions, whereby subjects lifted the object with their hand with their fingertips facing away from the body, subjects lifted the object during pre-rotation trails with their hand rotated around the object (as in the post-rotation trials of Condition 7), with their fingertips facing the body. The rationale for this particular pre-rotation trial procedure was based on results from the condition with the 180° rotation of the hand. For this condition (180° rotation of the hand), we found non-collinear digit placement on the first post-rotation trial (i.e. the thumb position was higher than the index fingertip position), regardless of whether the CoM was on the left or right side. We hypothesized that subjects partitioned their fingertips in this way due to the biomechanical constraints of the hand in this oriented position. With the digits facing the body and the wrist flexed, the non-physiological moment arms and/or the length of the finger flexors compared to their length-tension curve allows for supination to occur with greater ease than pronation. Supination would result in the thumb position being higher than index fingertip position. We tested this hypothesis by examining digit placement on the first pre-rotation trial when subjects had no explicit knowledge of the CoM location. Higher thumb than index finger placement on the first trial (compared to collinear digit placement seen when lifting a visually symmetrical object with the fingers facing away from the body) would support the hypothesis that subjects place their digits in this way due to biomechanical constraints. After the 8 pre-rotation trials, subjects were instructed to move 180°, toward the other side of the table, facing the object from the other side of the table, and lift the object with their fingertips facing away from the body. The procedure of the post-rotation trials was similar to that of the condition with the 180° rotation of the subject. Rotation of the subject and hand modified the body-to-object relation but not the hand-to-object relation from that during pre-rotation trials. For example, for an object with a right CoM, the right side of the body will be on the CoM side before rotation, and the left side will be on the CoM side after rotation, and the index finger will be on the CoM side before and after rotation.

### Data Analyses

Time of lift onset was defined as the time point at which the vertical position of the object exceeded 0.1 cm and continued to increase thereafter.

We measured peak object roll (in degrees) on the frontal plane of the object occurring after object lift onset, with positive and negative values denoting rolls in the direction of the thumb and the index finger, respectively.

We recorded digit load force, the vertical force component parallel to the grip surface exerted on the thumb and the index finger to lift the object, and computed the difference between these load forces. A zero value indicates symmetrical load forces by the thumb and the index finger, a positive value indicates that the thumb exerted more load force than the index finger, and a negative value indicates that the index finger exerted more load force than the thumb.

We also calculated the vertical coordinate of the point of resultant digit force relative to the center of the force-torque transducers (center of pressure). Center of pressure for the thumb and the index finger was defined as the vertical distance of each digit from the center of the grip surface/transducer (in mm), using the formula: COP = [M*x*–(F*tan* * *w*)] / F*n*], where M*x* is the moment about the x-axis (see Fig 1B), F*tan* is the digit load force, *w* is the distance between the surfaces of the force/torque transducer and the grip surface (4 mm), and F*n* is the mean grip force component perpendicular to the grip surface by the index finger and the thumb. Positive and negative values denote higher and lower center of pressure relative to the center of each transducer. We report the vertical distance between the centers of pressure of the index finger and the thumb (center of pressure difference). A zero value indicates collinear digit center of pressure, a positive value indicates that the thumb’s center of pressure is higher than that of the index finger, and a negative value indicates that the index finger’s center of pressure is higher than that of the thumb.

Finally, we calculated the compensatory moment (*N* mm) using the formula: M*com* = [(ΔF*tan*) * *d*/2 + F*n* * ΔCOP], where ΔF*tan* is the difference in load force between the thumb and the index finger, *d* is the grip width, and ΔCOP is the difference between the vertical coordinate of the thumb and index finger center of pressure. Positive and negative values denote compensatory moment generated in the direction of the index finger and thumb, respectively. Grip force (F*n*) can contribute to the compensatory moment, and thus roll, if the center of pressure difference between the thumb and the index finger is non-zero. However, we found no significant differences in grip force when comparing the trial preceding and following rotation for each of the conditions for left CoM and right CoM blocks, respectively (all *p’s* > 0.05).

Our main analyses focused on the effect of each rotation on object roll, compensatory moment, difference between the vertical coordinate of thumb and index finger center of pressure, and difference between the load force exerted by the thumb and index finger in each of the conditions for left and right CoM blocks, respectively. Therefore, we ran repeated measures analysis of variance (ANOVA) that examined the effect of trial (last pre-rotation trial, first post-rotation trial, and last post-rotation trial) on all the above variables. For significant main effects, we conducted Bonferroni-adjusted pairwise comparisons to examine differences between the first post-rotation trial and the last pre-rotation trial, and differences between the first post-rotation trial and the last post-rotation trial (setting the *p* value at 0.016 to adjust for 3 comparisons). We used the non-parametric McNemar’s test to examine the difference in direction of compensatory moment and the change in sign of center of pressure difference and load force difference between the last pre-rotation and the first post-rotation trial (see [18]). In addition, we qualitatively compared the extent to which subjects who gave incorrect responses in estimating the heavier side of the object performed differently to those that gave correct responses. Finally, to examine the extent to which learned manipulation transfer varies within conditions that modify object-hand-body relations (Conditions 5–8) and within those that do not modify these relations (Conditions 1–4), we compared roll on the first post-rotation trial within Conditions 5–8 and within Conditions 1–4, respectively, in each of the CoM blocks, using one-way ANOVAs.

## Results

We will describe the effect of each rotation in each of the 8 conditions on object roll, compensatory moment, center of pressure difference between the thumb and the index finger, and load force difference between the thumb and the index finger on the first post-rotation trial. Rotations in Conditions 1–4 (360° rotation of object, subject, or both, and 180° rotation of object and subject) maintained the relation between object and body, and between object and hand, from that during pre-rotation trials. Rotations in Conditions 5 and 6 (180° rotation of object and subject, respectively) modified the relations between object and body and between object and hand from that during pre-rotation trials. The rotation in Condition 7 (180° rotation hand rotation) modified the relation between object and hand, but maintained the relation between object and body, from that during pre-rotation trials. Finally, the rotation in Condition 8 (180° rotation of subject and hand) modified the relation between object and body from that during pre-rotation trials, but maintained the relation between object and hand. As described below, subjects in conditions with rotations that maintained the object-body and object-hand relations (Conditions 1–4) continued, after rotation, to generate effective compensatory moments to minimize roll by appropriate digit placement and load force distributions. Subjects in conditions with rotations that modified the relation between object and hand and/or between object and body (Conditions 5–8) did not generate effective compensatory moments and made large rolls after rotations, due to using the same, yet inappropriate digit placement and load force distributions as those used on pre-rotation trials.

### Maintaining the relation between object and hand and between object and body

**Fig 2:**
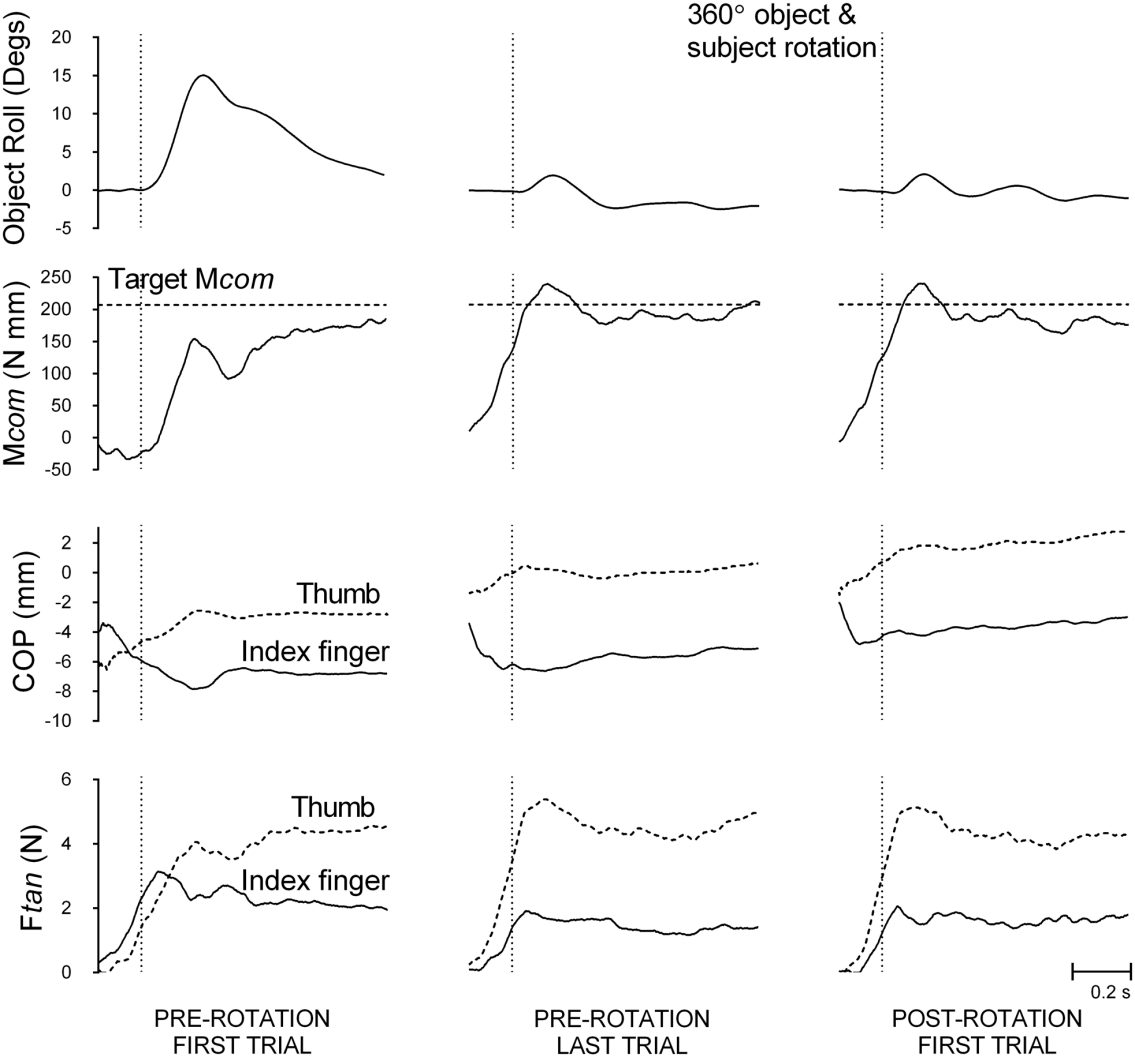
A representative subject’s performance traces in a condition that maintains object-subject and object-body relations. (A) Object roll; (B) Compensatory moment (M*com*, solid line) and target M*com* (dotted line, plotted as same sign as M*com* for graphical purposes); (C) center of pressure (COP) by the thumb (dotted line) and the index finger (solid line); (D) Load force (F*tan*) by the index finger (solid line) and the thumb (dotted line). Data are shown for the first (left panel) and last pre-rotation trial (middle panel), and following a rotation that does not modify the relation between the object and body, and object and hand (Condition 3), the first post-rotation trial (right panel), with the object’s CoM on the left. The vertical dotted line represents the lift onset time.

Fig 2 shows object roll, compensatory moment, and center of pressure and load force differences between the thumb and the index finger from a representative subject exposed to a rotation that did not modify the relation between object and body, and between object and hand from that in pre-rotation trials. Data are from the first and last pre-rotation trial with the subject lifting the object with a left CoM, and from the first post-rotation trial for Condition 3. On the first pre-rotation trial, having not experienced lifting the object, the subject lifted the object as if the CoM was centered in the visually-symmetric object. Specifically, the subject applied symmetrical load force in the thumb and the index finger and placed the thumb and the index finger collinearly. Consequently, the subject generated little or no compensatory moment, resulting in a large object roll to the left (the heavier side). This is the case for all subjects across conditions. However, by the last pre-rotation trial, the subject exerted larger load force in the thumb than the index finger, and placed the thumb higher than the index finger, which led to an effective compensatory moment that counteracted the external moment created by the CoM (toward the index finger), and minimized roll. This is all consistent with previous work [11,12,15,18,20,26]. Similar to the last pre-rotation trial, on the first post-rotation trial, the subject asymmetrically partitioned load force and digit placement by the thumb and the index finger, and thereby generated an effective compensatory moment to minimize object roll.

**Fig 3:**
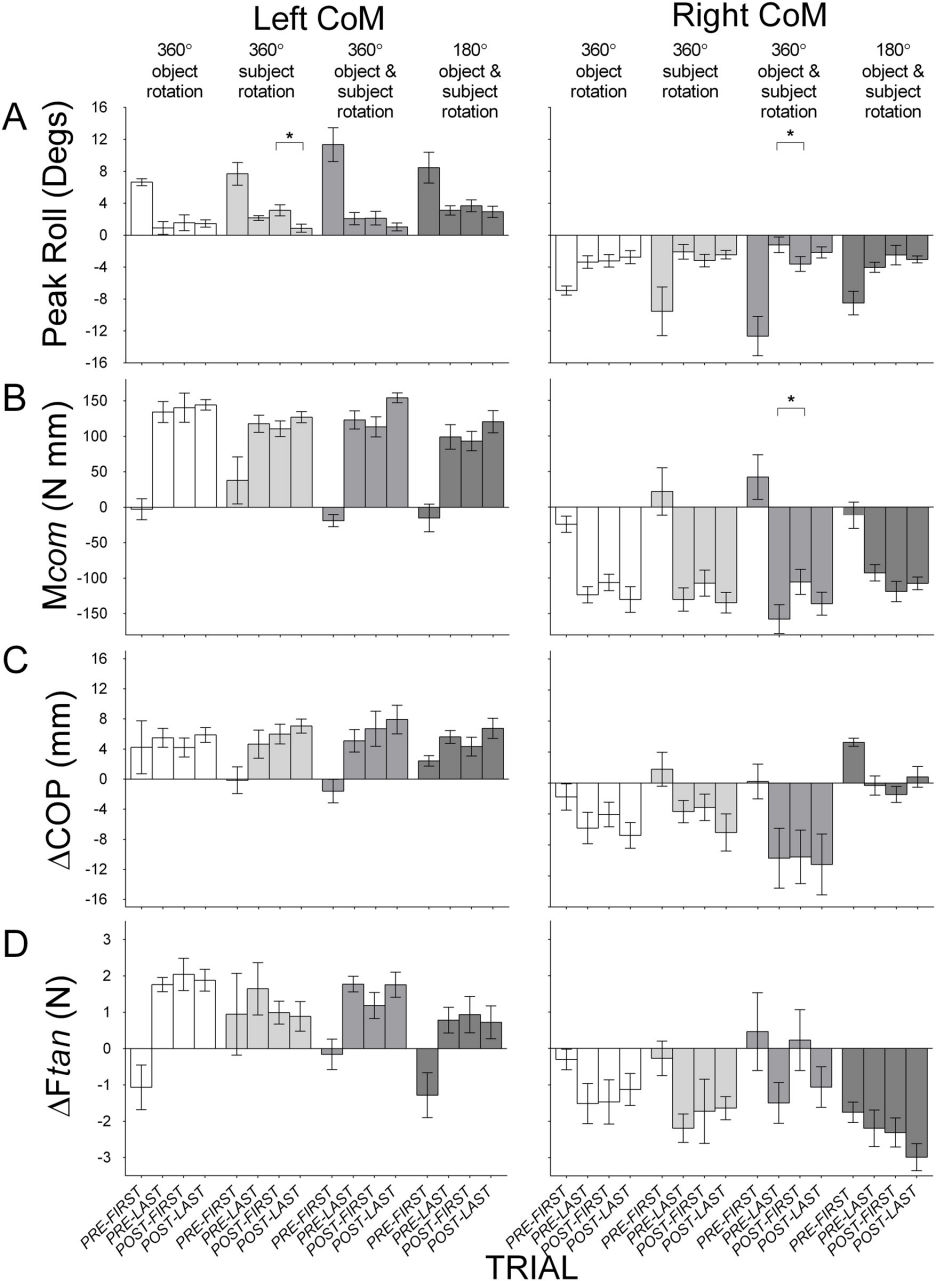
Group means (± 1 standard error) for Conditions 1–4 that maintain object-hand and object-body relations. (A) Object roll with positive and negative values indicating roll towards the thumb and the index finger respectively; (B) Compensatory moment (M*com*) with positive and negative values indicating moments generated away from the thumb and the index finger respectively; (C) Vertical distance between the thumb and the index finger center of pressure (ΔCOP) with positive values indicating higher thumb placement than index finger placement and negative values indicating higher index finger placement than thumb placement; (D) Difference in load force (ΔF*tan*) by the thumb and the index finger with positive values indicating more force by the thumb than the index finger and negative values indicating more force by the index finger than the thumb. Data are shown for the first and last pre-rotation trial, and the first and last post-rotation trial, with the object’s CoM on the left (left panel) and on the right (right panel) during pre-rotation trials, for Condition 1 (360° rotation of object; clear), Condition 2 (360° rotation of subject; light gray), Condition 3 (360° rotation of object and subject; medium gray) and Condition 4 (180° rotation of object and subject; dark gray). The first pre-rotation trial for the left and right CoM blocks in each condition is only shown for half of the subjects (because half started the task with the object’s CoM on the left and right, respectively). Statistically significant differences between the first post-rotation trial and the last pre-rotation and between the first post-rotation trial and the last post-rotation trial are denoted with an asterisk (*p* < 0.05).

Fig 3 shows that these findings were generally representative of all subjects in each of the four conditions (1–4) with rotations that did not modify the body-to-object and hand-to-object relations, and with the CoM of the object on the left and on the right. Other than very few exceptions that are described below, there were negligible differences in mean object roll, compensatory moment, digit center of pressure difference, and digit load force difference between the last pre-rotation trial and the first post-rotation trial, and between the first and the last post-rotation trial. There were no significant main effects of *Trial* (last pre-rotation, first post-rotation trial, last post-rotation trial) on object roll, compensatory moment, digit center of pressure difference, and digit load force difference in these conditions, except for the following: (1) object roll in Condition 2 (360° rotation of subject) when subjects lifted an object with a left CoM (*F*(2, 18) = 5.56, *p* < 0.05, η_p_^2^ = 0.38), with Bonferroni-adjusted pairwise comparisons showing a significant difference between the first and last- post-rotation trial, likely reflecting improvement with continual practice; (2) object roll (*F*(2, 18) = 5.96, *p* < 0.05, η_p_^2^ = 0.40) and compensatory moment (*F*(2, 18) = 6.11, *p* < 0.05, η_p_^2^ = 0.40) in Condition 3 (360° rotation of subject and the object) in the right CoM block, with significant differences between the last pre-rotation trial and the first post-rotation trials (however, there were no differences between the first and the last post-rotation trials, and the compensatory moment on both last pre-rotation and first post-rotation trials was in the same appropriate direction) and (3) digit center of pressure difference in Condition 1, 360° rotation of the object with a right CoM, (*F*(2, 18) = 4.26, *p* < 0.05, η_p_^2^ = 0.32), but no significant differences between the first post-rotation trial and both last pre- and post-rotation trials. Despite these differences in roll and compensatory moment, McNemar’s tests showed no significant differences in direction for compensatory moment and in sign for center of pressure difference and load force difference (*p’s* > 0.05) between the last pre-rotation trial and the first post-rotation trial in Conditions 1 to 4. In summary, these conditions predominantly showed no main effects of *Trial* on roll, compensatory moment, center of pressure difference, and load force difference, and no differences in direction and sign on these variables between pre- and post-rotation trials. This suggests that the act of rotating the object, subject, or both while maintaining the relation between object and body, and between object and digits, as that from trials preceding the rotation, does not disrupt the ability to transfer manipulation learned across trials preceding the rotation.

### Disrupting the relation between either object and hand or between object and body, or both

**Fig 4:**
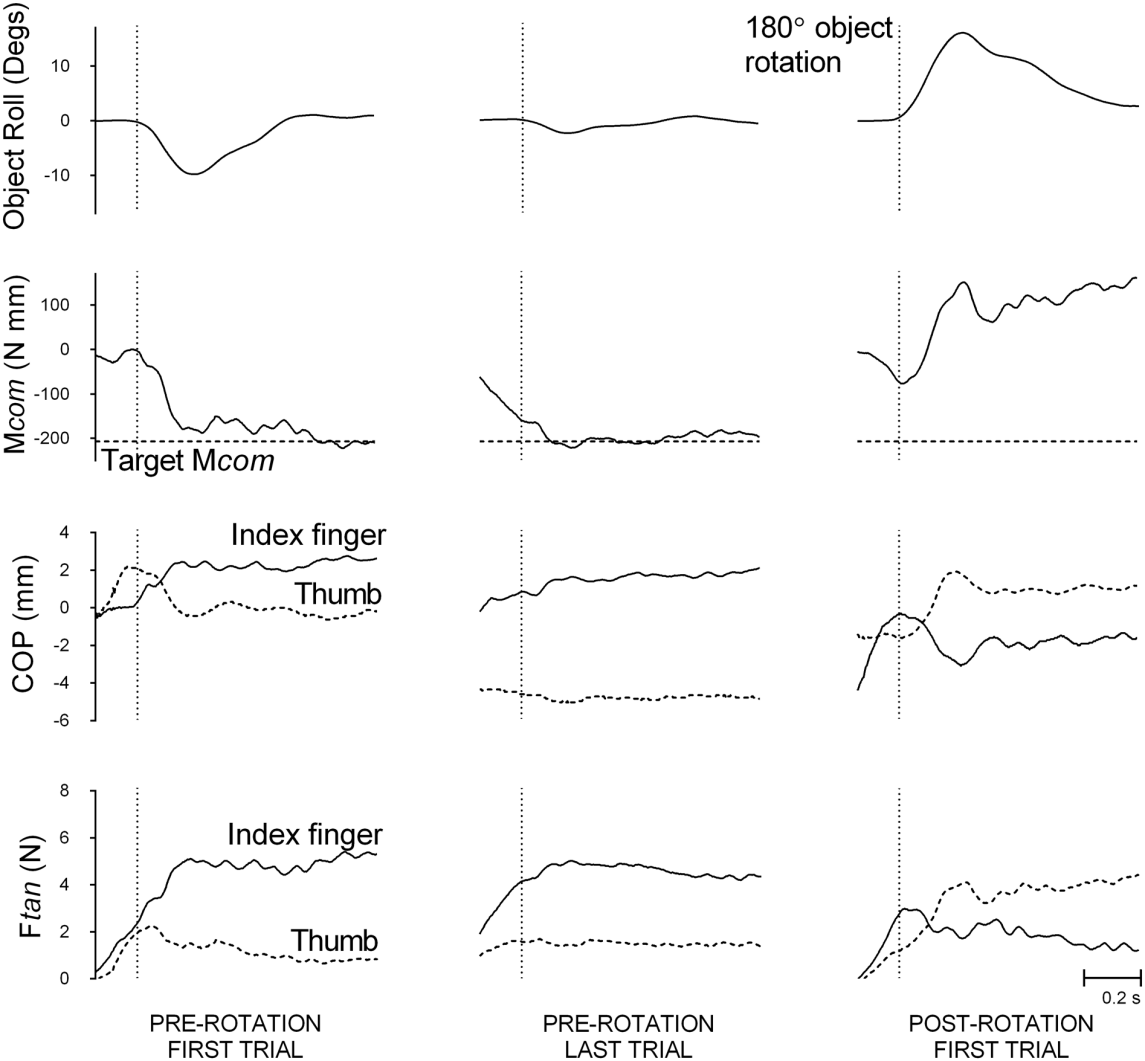
A representative subject’s performance traces by in a condition that modifies object-subject and object-body relations. (A) Object roll; (B) Compensatory moment (M*com*, solid line) and target M*com* (dotted line, plotted as same sign as M*com* for graphical purposes); (C) Center of pressure (COP) by the thumb (dotted line) and the index finger (solid line); (D) Load force (F*tan*) by the thumb (dotted line) and the index finger (solid line). Data are shown for the first (left panel) and last pre-rotation trial (middle panel) with the object’s CoM on the right and, following a rotation that modifies both the relation between the object and body and object and hand (Condition 5), the first post-rotation trial (right panel). The vertical dotted line represents the lift onset time.

Fig 4 shows data from a representative subject on object roll, compensatory moment, and center of pressure and load force differences between the thumb and index finger on the first and last pre-rotation trial and on the first post-rotation trial. The CoM is on the right during pre-rotation trials and on the left after 180° rotation of object. The relations between the object and subject’s body and between the object and subject’s hand were modified by this rotation. Similar to that seen in Fig 2, the thumb and the index finger load force was symmetrical and the thumb and the index finger center of pressure was collinear during the first pre-rotation trial, thereby resulting in negligible compensatory moment and large object roll. In contrast, during the last pre-rotation trial this subject exerted larger load force in the index finger than the thumb and placed the index finger higher than the thumb. Thus, this subject generated a large compensatory moment towards the thumb and minimized object roll accordingly. Ideally, to successfully minimize roll on the first post-rotation trial (with the CoM now shifted to the left), the subject should have applied larger load force and higher center of pressure by the thumb than the index finger, thereby generating a compensatory moment towards the index finger. In contrast to this ideal strategy, this subject continued to place his index finger higher than the thumb, and exerted larger load force by his index finger than the thumb on the first post-rotation trial. Thus, the compensatory moment was in the same, yet inappropriate, direction as that at the last pre-rotation trial, which resulted in large object roll. As described below, these findings were generally representative of subjects in conditions in which the rotation modified the relations between the object and body and/or between the object and hand, with a couple of nuisances in digit placement that are described below. We report below group data from each of the conditions that disrupt the relation between object and hand and object and body (Conditions 5 and 6) and conditions that disrupt the relation between object and hand (Condition 7) and object and body (Condition 8).

#### Disrupting the relation between object and hand and between object and body

**Fig 5:**
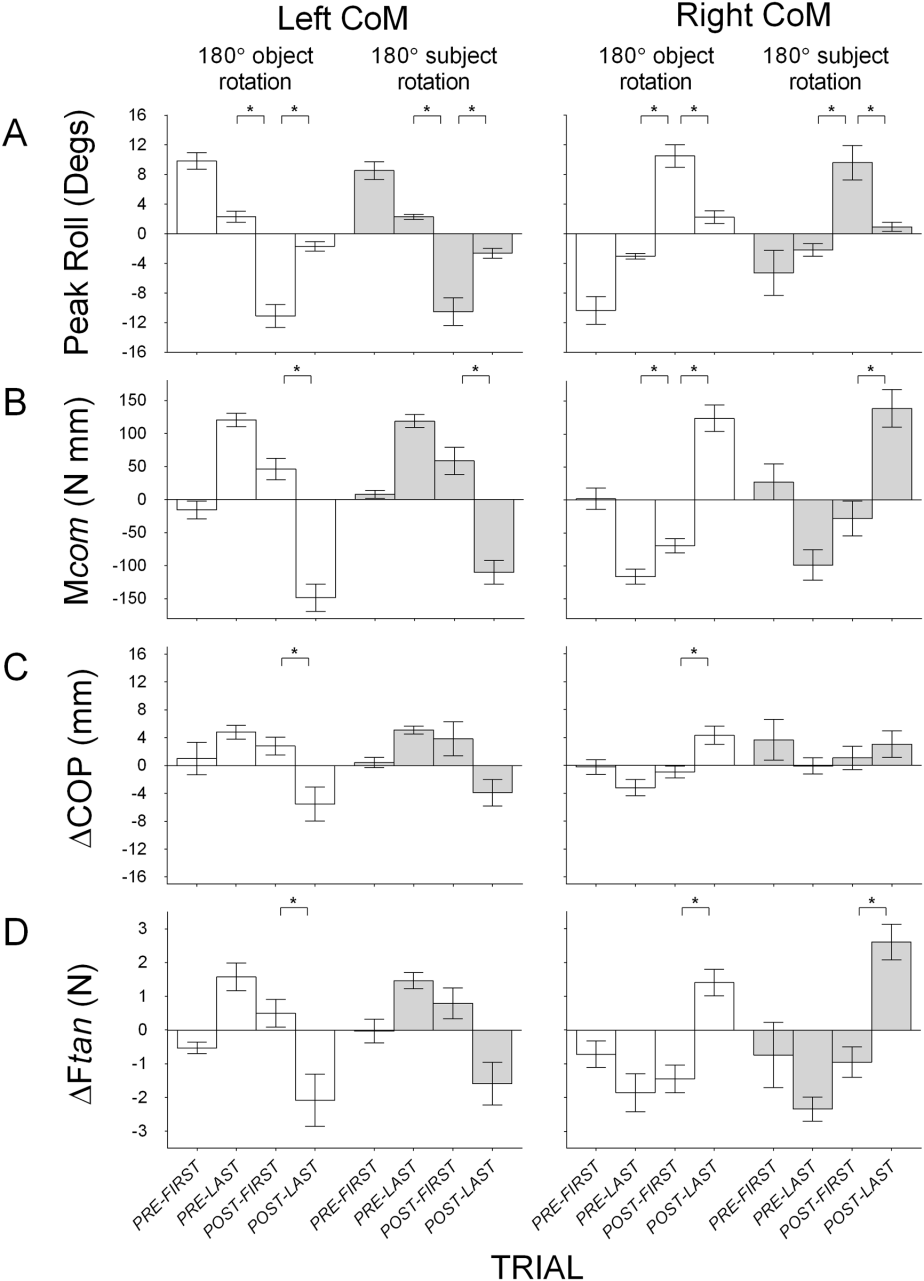
Group means (±1 standard error) for Conditions 5–6 that modify object-hand and object-body relations. (A) Object roll with positive and negative values indicating roll towards the thumb and the index finger respectively; (B) Compensatory moment (M*com*) with positive and negative values indicating moments generated away from the thumb and the index finger respectively; (C) Vertical distance between the thumb and the index finger center of pressure (ΔCOP) with positive values indicating higher thumb than index finger placement and negative values indicating higher index finger than thumb placement; (D) Difference in load force (ΔF*tan*) by the thumb and the index finger with positive values indicating more force by the thumb than the index finger and negative values indicating more force by the index finger than the thumb. Data are shown for the first and last pre-rotation trial, and the first last post-rotation trial, with the object’s CoM on the left (left panel) and on the right (right panel) during pre-rotation trials, for Condition 5 (180° rotation of object; clear) and Condition 6 (subject (180° rotation of subject; light gray). The first pre-rotation trial for the left and right CoM blocks in each condition is only shown for half of the subjects (because half started the task with the object’s CoM on the left and right, respectively). Statistically significant differences between the first post-rotation trial and the last pre-rotation and between the first post-rotation trial and the last post-rotation trial are denoted with an asterisk (*p* < 0.05).

Fig 5 shows mean peak roll, compensatory moment, center of pressure difference and load force difference between the thumb and the index finger on the first and last pre-rotation and on the first and last post-rotation trials, with the CoM of the object on the left and right for Conditions 5 (180° rotation of object) and 6 (180° rotation of subject). Compared to the last pre-rotation trial and the last post-rotation trial, subjects typically produced a large object roll and little compensatory moment on the first post-rotation trial, both of which were in the direction of the CoM. For both conditions and with the CoM on either side, there were significant main effects of *Trial* on roll (Condition 5 left CoM: *F*(2, 18) = 44.08, *p* < 0.05, η_p_^2^ = 0.83, right CoM: *F*(2, 18) = 45.40, *p* < 0.05, η_p_^2^ = 0.84; Condition 6 left CoM: *F*(2, 18) = 35.77, *p* < 0.05, η_p_^2^ = 0.80, right CoM: *F*(2, 18) = 16.24, *p* < 0.05, η_p_^2^ = 0.64) and compensatory moment (Condition 5 left CoM: *F*(2, 18) = 87.40, *p* < 0.05, η_p_^2^ = 0.91, right CoM: *F*(2, 18) = 86.82, *p* < 0.05, η_p_^2^ = 0.91; Condition 6 left CoM: *F*(2, 18) = 77.42, *p* < 0.05, η_p_^2^ = 0.90, right CoM: *F*(2, 18) = 17.53, *p* < 0.05, η_p_^2^ = 0.66), with very large effect sizes. Bonferroni-adjusted pairwise comparisons showed significantly larger object roll on the first post-rotation trial than both the last pre-rotation trial and the last post-rotation trial in both conditions. In the condition with the 180° rotation of object (with a right CoM during pre-rotation trials), compensatory moment was significantly smaller on the first post-rotation trial than both last pre- and last post-rotation trials. In the conditions with 180° rotation of object (with a left CoM during pre-rotation trials) and subject (with left CoM and right CoM during pre-rotation trials), compensatory moment was significantly smaller on the first post-rotation trial than the last post-rotation trial, but not the last pre-rotation trial. In addition, there was no significant difference in direction of compensatory moment between the last pre-rotation trial and the first post-rotation trial (*p* > 0.05). These findings suggest that these rotations disrupted the ability of subjects to transfer learned manipulation from trials preceding the rotation to the first trial after the rotation. Fig 5 also shows that subjects continued to use the same, yet inappropriate, digit placement and force distributions on the first post-rotation trial as on the last pre-rotation trial, or tended to use collinear digit placement. There were significant main effects of *Trial* on digit center of pressure difference (Condition 5 left CoM: *F*(2, 18) = 18.45, *p* < 0.05, η_p_^2^ = 0.67, right CoM: *F*(2, 18) = 10.71, *p* < 0.05, η_p_^2^ = 0.54; Condition 6 left CoM: *F*(2, 18) = 6.13, *p* < 0.05, η_p_^2^ = 0.41, right CoM: *p* > 0.05). Bonferroni-adjusted pairwise comparisons showed that there were no significant differences between the last pre-rotation trial and the first post-rotation trial for all conditions. We found, however, significant differences in digit placement between the first and last-post rotation trial (Condition 5, left and right CoM blocks). Finally, significant main effects of *Trial* on load force difference in all conditions with the CoM on either side (Condition 5 left CoM: *F*(2, 18) = 23.00, *p* < 0.05, η_p_^2^ = 0.72, right CoM: *F*(2, 18) = 13.92, *p* < 0.05, η_p_^2^ = 0.61; Condition 6 left CoM: *F*(2, 18) = 9.41, *p* < 0.05, η_p_^2^ = 0.51, right CoM: *F*(2, 18) = 32.49, *p* < 0.05, η_p_^2^ = 0.78) were due to differences between the first and last post-rotation trials (Conditions 5 left CoM and right CoM blocks, Condition 6, right CoM block). Taking center of pressure difference and load force difference results together, most of the main effects were due to significant differences between the first- and last post-rotation trials, with negligible differences between the last pre-rotation trial and the first post-rotation trial. This suggests that subjects adopted similar force and digit placement distributions on the first post-rotation trial as on the last pre-rotation trial, which were dissimilar to that used on the last post-rotation trial. Although in some cases there were negligible differences between the first and the last post-rotation trials, center of pressure difference and load force difference at the first post-rotation trial were in the same direction as those at the last pre-rotation trial in both conditions and CoM blocks (all *p’s* > 0.05). Together, these results suggest that rotating an object or subject in a way that modifies the relation between *both* object and body *and* object and hand, from that during pre-rotation trials disrupts the ability to transfer learned manipulation.

#### Disrupting the relation between object and hand while maintaining the relation between object and body

**Fig 6:**
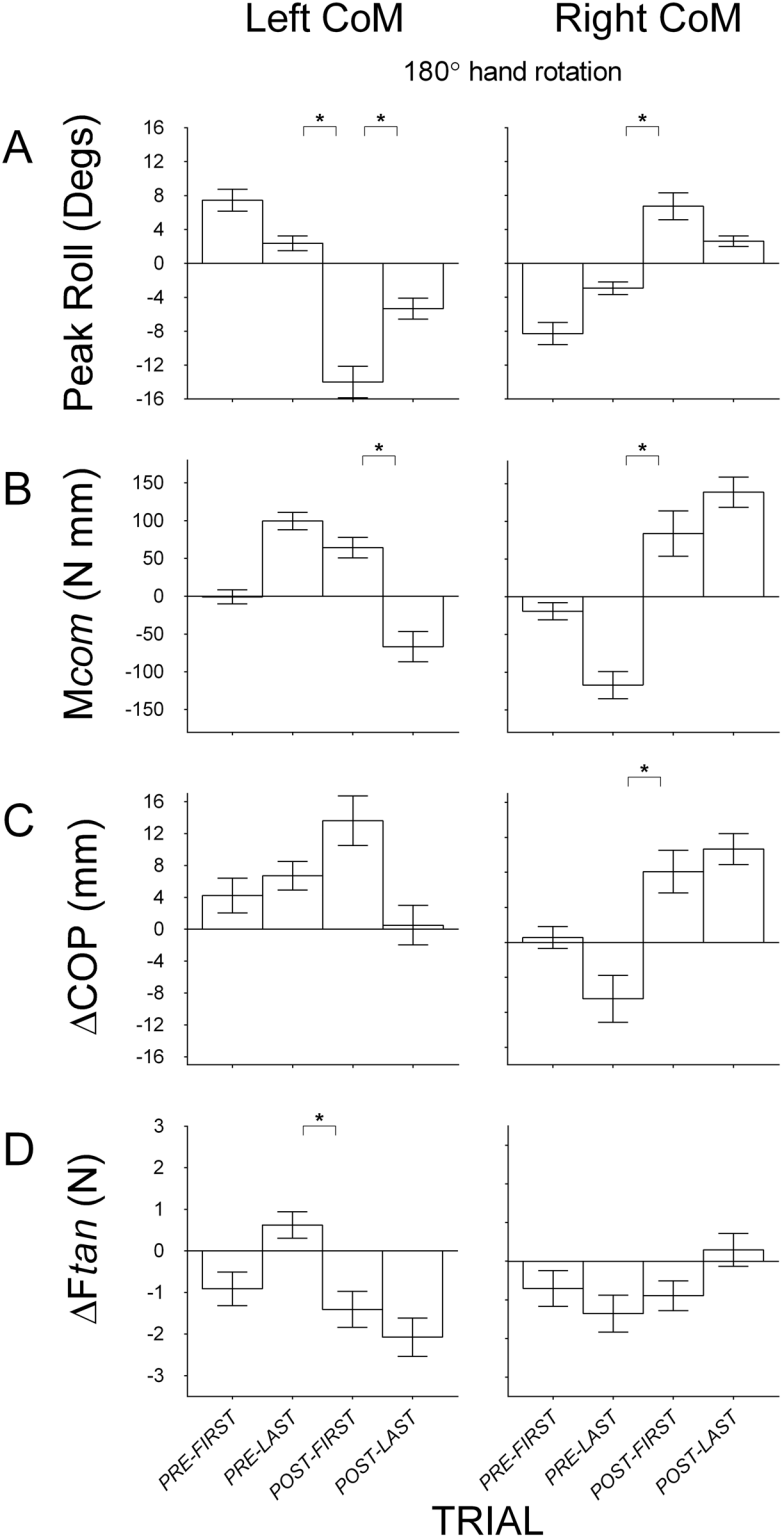
Group means (± 1 standard error) for Condition 7 that modifies the object-hand relation. (A) Object roll with positive and negative values indicating roll towards the thumb and the index finger respectively; (B) Compensatory moment (M*com*) with positive and negative values indicating moments generated away from the thumb and the index finger respectively; (C) Vertical distance between the thumb and the index finger center of pressure (ΔCOP) with positive values indicating higher thumb than index finger placement and negative values indicating higher index finger than thumb placement, and; (D) Difference in load force (ΔF*tan*) by the thumb and the index finger with positive values indicating more force by the thumb than the index finger and negative values indicating more force by the index finger than the thumb. Data are shown for the first and last pre-rotation trial, and for the first and last post-rotation trial, with the object’s CoM on the left (left panel) and on the right (right panel) during pre-rotation trials. The first pre-rotation trial for the left and right CoM blocks is only shown for half of the subjects (because half started the task with the object’s CoM on the left and right, respectively). Statistically significant differences between the first post-rotation trial and the last pre-rotation and between the first post-rotation trial and the last post-rotation trial are denoted with an asterisk (*p* < 0.05).

Fig 6 shows mean peak roll, compensatory moment, center of pressure and load force differences between the thumb and the index finger at the first and last pre-rotation trial, the first and last post-rotation trial, in both left and right CoM blocks for Condition 7 (180° rotation of hand). As the figure shows, on the first trial after hand rotation with the CoM on the left (index finger side), subjects placed the thumb higher than the index finger, and exerted more load force with the index finger than the thumb. The combined effect of these responses resulted in negligible compensatory moment and large object roll. This particular configuration of load force and center of pressure by the index finger and the thumb was also seen on the first post-rotation trial with the CoM of the object on right (thumb side). In both CoM blocks, compensatory moment was generated towards the index finger, which is the appropriate direction when the object’s CoM is on the right, but not the left. Nevertheless, as Fig 6 shows, subjects produced large rolls on this first post-rotation trial in both CoM blocks, albeit smaller when the CoM was on the right. We found significant main effects of *Trial* on roll for both left CoM (*F*(2, 18) = 30.90, *p* < 0.05, η_p_^2^ = 0.77) and right CoM blocks (*F*(2, 18) = 15.32, *p* < 0.05, η_p_^2^ = 0.63) with large effect sizes. Pairwise comparisons showed significant differences in roll between the first post-rotation trial and the last pre-rotation trial in both CoM blocks, but a significant difference between the first and last post-rotation trial only for the left CoM block. This suggests that subjects minimized roll on the first post-rotation trial similarly to that on the last post-rotation trial in the right CoM block, but failed to do so in the left CoM block. We found significant main effects of *Trial* on compensatory moment when the object CoM was on the left (*F*(2, 18) = 36.09, *p* < 0.05, η_p_^2^ = 0.80) and right (*F*(2, 18) = 28.12, *p* < 0.05, η_p_^2^ = 0.76) with large effect sizes. Pairwise comparisons showed significant difference between the first and last post-rotation trial, but not the last pre-rotation trial, for the left CoM block, and a significant difference between the first post-rotation trial and last pre-rotation trial, but not the last post-rotation trial, for the right CoM block. Again, this indicates the compensatory moment on the first post-rotation trial was similar to that on the last post-rotation trial in the right CoM block, but not the left CoM block. In addition, a significant difference in direction between the last pre-rotation trial and the first post-rotation trial in the right CoM block (*p* < 0.05) but not in the left CoM block (*p* > 0.05) suggests that subjects transferred learned manipulation after hand rotation to a greater extent when the object’s CoM was on the right than the left. We found a significant main effect of *Trial* on load force difference for the left CoM block with a large effect size (*F*(2, 18) = 22.69, *p* < 0.05, η_p_^2^ = 0.72). Pairwise comparisons showed a significant difference between the last pre-rotation trial and the first post-rotation trial (and a significant change in sign, *p* < 0.05), and no significant main effect of trial on load force difference for the right CoM block (and no change in sign, *p* > 0.05). We also found significant main effects of *Trial* on center of pressure difference for the left (*F*(2, 18) = 5.58, *p* < 0.05, η_p_^2^ = 0.38) and right CoM blocks (*F*(2, 18) = 12.81, *p* < 0.05, η_p_^2^ = 0.59) with large effect sizes. In addition, pairwise comparisons showed a significant difference between the first post-rotation trial and the last pre-rotation trial for the right CoM block (and with the sign only changing for the right, but not left block), and no difference between the first and last post-rotation trial for both CoM blocks.

In summary, we show differing results for left and right CoM blocks. Subjects failed to minimize roll on the first post-rotation trial more so when lifting an object with a left than a right CoM when the hand is oriented around the object, all of which might be a function of the biomechanical constraints of the hand in this orientation. Higher positioning of the thumb than index finger instead of collinear digit placement on the very first pre-rotation trial with the hand rotated around the object (when subjects have no knowledge of the asymmetric CoM in the visually symmetric appearing object) would support the hypothesis that biomechanical constraints of the hand in this orientation contributes to this particular digit partitioning, and thus compensatory moment and object roll results obtained in Condition 7. We report our examination of this hypothesis in the next section.

#### Disrupting the relation between object and body while maintaining the relation between object and hand

**Fig 7:**
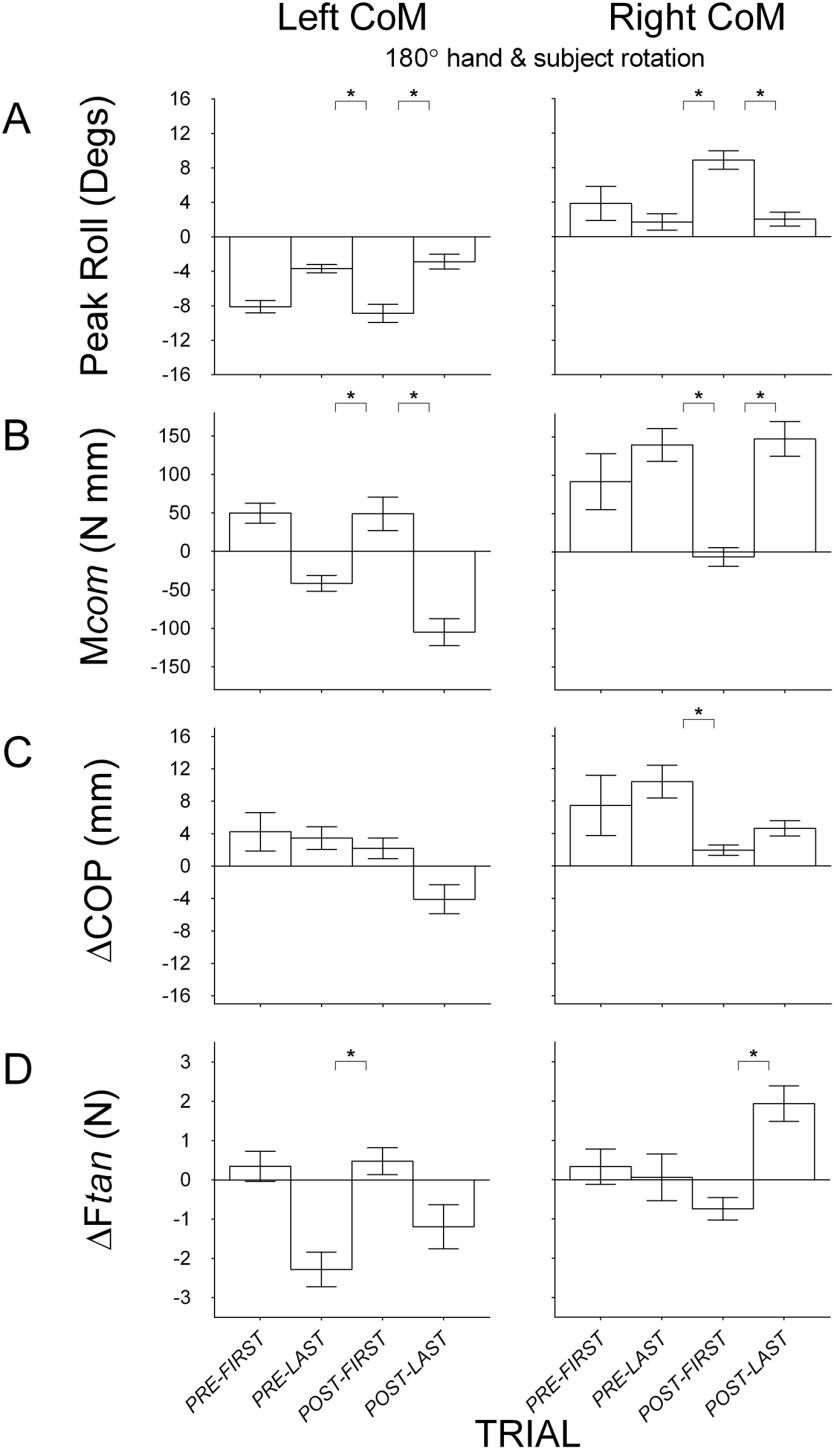
Group means (± 1 standard error) for Condition 8 that modifies the object-body relation. (A) Object roll with positive and negative values indicating roll towards the thumb and the index finger respectively; (B) Compensatory moment (M*com*) with positive and negative values indicating moments generated away from the thumb and the index finger respectively; (C) Vertical distance between the thumb and the index finger center of pressure (ΔCOP) with positive values indicating higher thumb than index finger placement and negative values indicating higher index finger than thumb placement; (D) Difference in load force (ΔF*tan*) by the thumb and the index finger with positive values indicating more force by the thumb than the index finger and negative values indicating more force by the index finger than the thumb. Data are shown for the first and last pre-rotation trial, and for the first and last post-rotation trial, with the object’s CoM on the left (left panel) and on the right (right panel) during pre-rotation trials. The first pre-rotation trial for the left and right CoM blocks is only shown for half of the subjects (because half started the task with the object’s CoM on the left and right, respectively). Statistically significant differences between the first post-rotation trial and the last pre-rotation and between the first post-rotation trial and the last post-rotation trial are denoted with an asterisk (*p* < 0.05).

In this condition (Condition 8), subjects lifted the object on the pre-rotation trials with the hand rotated around the object such that the fingertips faced the body, and following the pre-rotation trials, moved to the other side of the table and lifted the object such that the fingertips faced away from the body. The object CoM was on the same digit-side on pre and post-rotation trials. Fig 7 shows mean object roll, compensatory moment, center of pressure and load force by the index finger and the thumb, on the first and last pre-rotation trial, and on the first and last post-rotation trials of Condition 8. On the first post-rotation trial, compared to the last pre-rotation trial and the last post-rotation trial, object roll was larger and the compensatory moment was in the opposite (*p*’s < 0.05), and incorrect, direction in both CoM blocks. With significant main effects of *Trial* on roll (left CoM: *F*(2, 18) = 15.86, *p* < 0.05, η_p_^2^ = 0.64; right CoM: *F*(2, 18) = 43.51, *p* < 0.05, η_p_^2^ = 0.83) and compensatory moment (left CoM: *F*(2, 18) = 23.15, *p* < 0.05, η_p_^2^ = 0.72; right CoM: *F*(2, 18) = 21.78, *p* < 0.05, η_p_^2^ = 0.71), pairwise comparisons showed significant differences between the first post-rotation and both the last pre-rotation trial and the last post-rotation trial. Fig 7 shows positive center of pressure difference values in both left and right CoM blocks on the first and last pre-rotation trial, indicating higher thumb than index placement. The higher placement of the thumb than index finger on the first and last pre-rotation trial in the left CoM block, while the index finger is on the heavier side of the object, further supports the hypothesis for this particular digit placement configuration and hand orientation to be a function of the biomechanical constraints of the hand. We found significant main effect of *Trial* on center of pressure difference in the left (*F*(2, 18) = 7.40, *p* < 0.05, η_p_^2^ = 0.45) and right CoM blocks (*F*(2, 18) = 13.24, *p* < 0.05, η_p_^2^ = 0.60). However, there were no significant differences between the first post-rotation trial and both last pre-rotation trial and last post-rotation trial in the left CoM block. In the right CoM block, we found a significant difference between the first post-rotation trial and the last pre-rotation trial, and no difference between first and last post-rotation trials. Finally, load force difference, as shown in Fig 7, was typically smaller on the first post-rotation trial than the last post-rotation trial (both CoM blocks) and the last pre-rotation trial (left CoM block only). We found significant main effects of *Trial* on load force difference for both left (*F*(2, 18) = 7.93, *p* < 0.05, η_p_^2^ = 0.47) and right CoM blocks (*F*(2, 18) = 8.91, *p* < 0.05, η_p_^2^ = 0.50). Pairwise comparisons showed significant differences between the first post-rotation trial and the last post-rotation trial for the right CoM block, and a significant difference between the first post-rotation trial and the last pre-rotation for the left CoM block. Although there were some significant differences between the last pre-rotation trial and the first post-rotation trial in center of pressure difference (right CoM block) and load force difference (left CoM block), McNemar’s tests showed no significant change in sign between the last pre-rotation trial and the first post-rotation trial for either of these variables in either CoM blocks. Taken together, the results suggest that modifying the relation between the object and the body while maintaining the relation between the object and digits disrupts the ability to transfer manipulations learned in the trials preceding the rotation.

### Comparing object roll following rotation in subjects who correctly and incorrectly estimated CoM-side of the object

As indicated above, we excluded 7 subjects who were unable to verbally indicate the side of the object that was heavier following the last pre-rotation trial. If we included these subjects, we could not have ruled out that failed learning transfer following rotation in any of the conditions was due to not having explicit knowledge of the CoM location. Surprisingly, the mean object roll on the first post-rotation trial by subjects within Conditions 1 to 4 who gave an incorrect estimate of CoM location in both left (*n* = 3; *M* = 2.27, *SD* = 1.91) and right CoM blocks (*n* = 3; *M* = -3.08, *SD* = .39) was within the standard deviation of the mean object roll by subjects within the same conditions who gave correct CoM-side estimates(Conditions 1–4: left CoM block: *M* = 2.62, *SD* = 2.64, right CoM block: *M* = -3.13, *SD* = 2.91), respectively. Similarly, object roll on the first post-rotation trial by the one subject in Condition 6 who gave an incorrect CoM-side estimate in the left CoM block (*M* = -15.03) was also within the standard deviation of the mean object roll by subjects in the same condition and block who gave correct CoM-side estimates (*M* = -10.52, *SD* = 5.86). Thus, the inability to explicitly identify the heavier side seemingly did not affect task performance.

### Comparing learning transfer within conditions that modify and within conditions that maintain relations between object, hand, and body

As the above results showed, any rotations that disrupted the relation between object and body, object and hand, and both, impaired the ability of subjects to successfully minimize roll on the first post-rotation trial whereas rotations that maintained these relations gave no such impairments. We compared object roll on the first post-rotation trial within Conditions 5–8, which modified the relation between object and hand, or object and body, or both. We also compared object roll on the first post-rotation trial within Conditions 1 to 4, which maintained the relations between object and hand and object and body. In both comparisons, we found no significant differences in object roll on the first post-rotation trials within Conditions 1 to 4 and within Conditions 5 to 8, for left and right CoM blocks, respectively (all *p*’s > 0.05). These findings suggest that disruption to any relation (object and body, object and hand, both) in which an object manipulation task is learned will give way to impaired performance of similar magnitudes.

## Discussion

We examined the ability to minimize roll of an object with an asymmetric mass distribution during a grasp and lift task, followed by rotations that either maintained (Conditions 1–4) or modified (Conditions 5–8) the relation between the object and the body, hand, or both, from that preceding the rotation. This task required modulating compensatory moment, through a combination of asymmetric partitioning of digit position and load force by the thumb and the index finger, to minimize object roll. Subjects in Conditions 1 to 4 generated an appropriate compensatory moment to minimize roll on the last pre-rotation and on the first post-rotation trial. Therefore, there was a transfer in learned compensatory moment to minimize roll following rotations in Conditions 1 to 4. In contrast, and as hypothesized, following rotations in Conditions 5 to 8, there generally were large differences in the ability to transfer learned compensatory moment and therefore minimize roll on the first post-rotation trial. Specifically, subjects produced large object rolls compared to the last pre- and the last post-rotation trials. Together, these findings extend those of previous studies in two important ways. First, successful transfer of learned manipulation following rotations in Conditions 1 to 4 suggests that failed transfer of learning following rotations in Conditions 5 to 8 is not an artifact of having visually observed and/or performed a rotation. Second, failed learning transfer also occurs (and of similar magnitude) when modifying either object-to-body or object-to-hand relations compared to modifying both relations. This suggests that modifying one reference frame is no more detrimental to grasp performance than another. As described below, these findings support the notion that internal representations of learned manipulations of objects with asymmetric mass distributions are specific to the context and reference frames in which they were learned and, therefore, that multiple representations exist for sensorimotor control of the hand [22,23].

Previous studies that investigated the effect on grasp performance of rotating objects with asymmetric mass distributions did not consider an alternative explanation for failed learning transfer. Specifically, given the complexities involved in mental rotation, any kind of rotation (even that which maintains body and hand frames) might disrupt the ability to minimize roll. Here we show that subjects could transfer learned manipulation following rotations that maintain hand and body frames relative to the object, even in conditions whereby the orientation of the object is changed from that prior to the rotation (180° rotation of object and subject). These findings suggest that the rotation experienced or performed, which subjects were asked to attend to (by watching the object being rotated, or watching the object as they moved around it), did not disrupt their ability to successfully retrieve and use the internal representation formed during trials preceding the rotation. The fact that experiencing a rotation was not detrimental to grasp performance in Conditions 1 to 4 suggests that the disruption in grasp performance following rotations in Conditions 5 to 8 is unlikely a byproduct of observing or performing a rotation. Thus, our findings support the notion that internal representations of learned manipulations with objects with asymmetric mass distributions are specific to the hand and body frames (relative to the object) in which the manipulation was first learned.

The fact that in Conditions 5 to 8 subjects generally failed to counteract the external moment on the object and thus to prevent object roll in the direction of the CoM following 180° rotation of object, subject, hand, and hand and subject, are consistent with findings from previous object rotation and hand translation studies [11,12,13,15,18,20,21]. Specifically, this previous work and our findings showed that failure to prevent object roll on the first post-rotation trial is due to inappropriate scaling of forces [11,12,13,15,21] as well as inappropriate digit placement [18,20]. Inspection of digit and force partitioning by the thumb and the index thumb following rotations of object, subject, hand, and hand and subject (see Figs 4 through 7), as well as results from McNemar’s tests, indicate that subjects in these conditions typically followed the same, yet inappropriate, digit and force partitioning pattern following rotation as that prior to rotation. There were some exceptions, where subjects reverted to a “default” digit force-position pattern following rotation, whereby the object was treated as having a symmetrically distributed mass, e.g. applying the same load force by index finger and thumb—see Fig 7, left CoM. The phenomenon of implementing the same motor output following a rotation perturbation is reminiscent of that seen in reaching studies. Specifically, after adapting to Coriolis forces [27] viscous forces [28,29,30,31], inertial loads [32], or rotations of visual feedback about the hand [33,34,35,36,37], subjects continued to use the same motor command and adopted the same movement strategies, thereby resulting in aftereffects. As has also been shown in visuomotor reaching studies [36], one might expect learning transfer to fall off continuously as a function of the magnitude of the rotation relative to the training direction (that is, if the same mechanism is shared across our task and learning of reaching movements). Our present findings suggest that such deterioration in learning transfer might be a function of the change in body-to-object relation associated with the increase in angle from the training direction. We did not test rotations less than 180° as they would necessitate maintaining the hand-object relation and/or placing the hand in a biomechanically awkward position after rotation. However, it should be noted that even after 360° rotations, subjects could successfully transfer learned manipulation. Thus, we conclude it is unlikely that our findings are dependent on the magnitude of the rotation relative to the trained direction.

Our findings support the conclusions of a study by [22] that examined how the experience with the dynamics of a specific hammer in one orientation generalized to other orientations. The aim of [22]’s study was to test two alternative hypotheses about whether the motor system used multiple representations of the dynamics associated with different tool orientations or, conversely, whether the motor system used a single general representation that applied to all virtual tool orientations. These authors argued that transfer of learning in one orientation to a novel orientation would support the notion of a single representation applying to all orientations, whereas limited transfer to a novel orientation would support the existence of multiple orientation-specific representations. Their results support the latter hypothesis as there was limited learning transfer when the hammer was presented in a novel orientation relative to the one subjects learned the manipulation in. In addition, and consistent with our findings, [22] showed that subjects were not using a default force pattern in this new orientation, but were using the same force pattern as that used during the training orientation. Their results and ours suggest that internal representations of object manipulation are orientation specific, and that multiple representations exist for sensorimotor control, with the appropriate representation being selected based on the context in which the movement occurs [22,23,38,39,40]. Furthermore, when a mismatch occurs in the reference frame between the learned orientation (in which an object manipulation task is learned) and a novel orientation, the manner with which an object is first grasped in a novel orientation (with regards to forces and position) generally mimics the manner with which it was grasped in the preceding orientation. This is at least the case during the early stages of sensorimotor learning.

The nature of internal representations relating to previous experiences with objects with asymmetric mass distributions is not well understood. For example, it is unknown whether the reference frame of early formed representations in one orientation is, relative to the object, specific to the hand, the body, or both, because rotations used by studies to date (mostly 180° rotation of object) modified both relations between the object and body and between the object and hand. In the present experiments, we included conditions that either modified the relation between object and body, or between object and hand, or both, to determine if the same magnitude of disruption to grasp performance would occur in each of these conditions. This was found to be the case. From these findings, it could be that the internal representation is not only specific to the hand (and digits) with which the task was learned, but the representation is also specific to the body position (relative to the object) in which the task was learned. Disrupting either the relation between the object and digits, or between the object and body position, will give way to deteriorated grasp performance of similar magnitude. However, there might be another contributing factor that for failed learning transfer in Condition 7 (hand rotation) and Condition 8 (hand and subject rotation), the conditions which disrupted either hand or body frames, respectively. Rotations in both these conditions not only changed the hand position relative to the object (Condition 7) or the body position relative to the object (Condition 8). The rotations in these conditions also changed the hand position relative to the body (from the fingers pointing away from the body prior to rotation to the fingers pointing toward the body following rotation in Condition 7, and vice versa in Condition 8). Thus, another factor that could explain failed transfer of learned manipulation is that the learned representation is also specific to the hand position relative to the body position. If this is the case, then the learned representation, which is specific to the hand and body relation in which it was formed, cannot be used to successfully grasp the object when the map between hand and body changes.

A study by [15] included a condition similar to our Condition 7, which involved rotation of the hand around the object. They reported learning transfer following rotation of the hand, which is in line with what we found in the right CoM block, but not the left CoM block. The results from Condition 7 (right CoM block) and that from [15] are in contrast to all other findings from conditions with rotations that modified hand and/or body frames from that prior to the rotation. When the object’s CoM was on the right, and following a rotation of the hand around the object, roll was in the direction of the CoM but not significantly larger than that on the last trial following rotation. In addition, unlike all other findings from conditions with modified hand or body frames (or both), compensatory moment was in the same direction at the first and last post-rotation trial. From inspection of Fig 6, it seems that this appropriate behavior on the first trial following rotation was a result of higher thumb than index finger placement when the hand was oriented around the object. The fact that the same digit partitioning was seen in both CoM blocks following hand rotation led to our hypothesis that such behavior could have been due to the biomechanical constraints imposed on the hand when placed in this particular orientation. Similar asymmetrical digit partitioning on the first trial of Condition 8 (when the hand was also oriented around the object) supported our hypothesis that this particular digit placement configuration and hand orientation was a function of the biomechanical constraints of the hand. Interestingly, in both CoM blocks on the first post-rotation trial of Condition 7, subjects exerted more load force with the index finger than the thumb. This phenomenon, too, could be confounded by biomechanical factors, i.e. higher thumb than index placement might have to be accompanied by higher load force by the index finger than the thumb to prevent the object from slipping. The biomechanical constraints of the hand with the fingertips oriented toward the subject can similarly explain the results from [15] study. They combined findings from lifting an object with a left CoM and right CoM, respectively, such that it was not possible to see whether differences were seen between these different blocks. Nevertheless, since they do not report forces and did not measure digit position, it is unknown whether subjects exhibited the same digit force and position partitioning on the first post-rotation trial as what was shown here. Thus, we conclude that the likely contribution of biomechanical constraints of the hand in this orientation to behavioral results of our Condition 7 (appropriate behavior in the right CoM block but inappropriate behavior in the left CoM block), and of [15], cannot be ruled out.

Finally, the finding that subjects who were unable to articulate explicit knowledge of the CoM faired no worse in minimizing object roll than subjects who could do so is consistent with the proposition that consecutive exposure to manipulations of an object with a given CoM location allows for *implicit* learning about the magnitude and direction of the external torque caused by the added mass [18]. Furthermore, this result suggests that explicit knowledge of the object CoM was not necessary for successful grasp performance in this subset of subjects. However, it remains to be determined why this mismatch between implicit and explicit knowledge of object CoM occurred only in this subset of subjects.

In summary, our findings extend those of previous object rotation studies by showing failed transfer of learning following rotation is not simply an artifact of having visually observed or performed a rotation. Furthermore, our findings suggest that internal representations of an object with an asymmetric CoM are orientation-specific, and that there are multiple representations for manipulating these objects in multiple orientations. These internal representations can be retrieved and used to successfully manipulate an object only when the reference frame in which the manipulation was learned matches the reference frame in which the manipulation is performed, at least during the early stages of sensorimotor learning.

## Supporting Information

S1 File Individual data points for all subjects in each of the 8 conditions. (XLSX)
